# Environmental Filtering of Microbial Communities in Agricultural Soil Shifts with Crop Growth

**DOI:** 10.1371/journal.pone.0134345

**Published:** 2015-07-30

**Authors:** Sarah K. Hargreaves, Ryan J. Williams, Kirsten S. Hofmockel

**Affiliations:** Department of Ecology, Evolution and Organismal Biology, Iowa State University, Ames, IA, United States of America; Netherlands Institute of Ecology (NIOO/KNAW), NETHERLANDS

## Abstract

Plant and soil properties cooperatively structure soil microbial communities, with implications for ecosystem functioning. However, the extent to which each factor contributes to community structuring is not fully understood. To quantify the influence of plants and soil properties on microbial diversity and composition in an agricultural context, we conducted an experiment within a corn-based annual cropping system and a perennial switchgrass cropping system across three topographic positions. We sequenced barcoded *16S* ribosomal RNA genes from whole soil three times throughout a single growing season and across two years in July. To target the belowground effects of plants, we also sampled rhizosphere soil in July. We hypothesized that microbial community α-diversity and composition (β-diversity) would be more sensitive to cropping system effects (annual vs. perennial inputs) than edaphic differences among topographic positions, with greater differences occurring in the rhizosphere compared to whole soil. We found that microbial community composition consistently varied with topographic position, and cropping system and the rhizosphere influenced α-diversity. In July, cropping system and rhizosphere structured a small but specific group of microbes implying a subset of microbial taxa, rather than broad shifts in community composition, may explain previously observed differences in resource cycling between treatments. Using rank abundance analysis, we detected enrichment of Saprospirales and Actinomycetales, including cellulose and chitin degraders, in the rhizosphere soil and enrichment of Nitrospirales, Syntrophobacterales, and MND1 in the whole soil. Overall, these findings support environmental filtering for the soil microbial community first by soil and second by the rhizosphere. Across cropping systems, plants selected for a general rhizosphere community with evidence for plant-specific effects related to time of sampling.

## Introduction

Soil microorganisms mediate biogeochemical transformations that underpin important ecosystem functions. Bacteria and fungi decompose organic matter and mineralize it to plant available forms [1], retain nutrients [2], and influence atmospheric concentrations of greenhouse gases [3]. Thus, for agricultural ecosystems, the response of soil microorganisms to environmental parameters has important implications for crop productivity and the long-term suitability of a soil to agriculture. There is evidence that soil microbial community function can be influenced by community structure [4–7], and that both are cooperatively shaped by plants and soil properties [8,9]. There are contrasting reports in the literature, however, as to the relative contribution of plant and soil properties as factors structuring microbial community composition and diversity.

Edaphic factors are thought to be an important environmental filter shaping soil microbial communities, including the microbial seed back, at a range of scales [8]. For example, bacterial community composition and diversity are shaped largely by soil pH at relatively coarse scales [10,11]. At finer scales, pH along with soil texture and nutrient status can shape soil microbiota [12]. Within an individual soil, the quantity and chemistry of root exudates and plant residues can stimulate or inhibit different microbial taxa, resulting in distinct microbial communities associated with specific plants [13–15]. Differences in root production and phenology can alter resource availability in time and space, and thus also structure microbial communities [16]. In addition to patches of plant residues [17], a hotspot for a plant’s influence on microbial communities is the rhizosphere, the narrow zone of soil that surrounds and is influenced by plant roots [8,18]. Here, microbial communities are enriched from the whole soil in response to the availability of low-molecular-mass compounds and polymerized sugar from root cells. Additionally, plant secondary metabolites are involved in establishing symbioses and repelling pests and pathogens, and root water and nutrient uptake alter pH and resource availability for microbes [8]. These influences illustrate the role that the rhizosphere plays as a second environmental filter on soil microbiota, with cascading effects to biogeochemical cycling of carbon (C) and nitrogen (N) [19].

In agricultural ecosystems, management practices add another level of complexity to the edaphic forces structuring soil microbial communities. Management influences microbial communities directly by altering soil properties, and indirectly through changes in plant nutrient and water requirements. One management action common across diverse types of agriculture (e.g. pasture, row-crop) and cropping systems (e.g. annual- and perennial-based bioenergy crops) is N enrichment from fertilization. Nitrogen fertilization, especially from inorganic sources, can affect soil microbiota directly by increasing N availability for microbes [20], and indirectly by altering plant-derived C inputs to soils [21,22] and increasing soil acidity [23,24]. These microbial shifts, in turn, may alter rates of decomposition [20] and weaken plant-microbe linkages critical to ecosystem nutrient retention [25].

In a previous study contrasting cropping system and topographic position as a proxy for edaphic factors, cropping system had a stronger influence on the functional capacity of the microbial community (potential enzyme activity, net N mineralization and respiration) than edaphic factors associated with topographic position [26]. We did not observe changes in microbial activity concomitant to changes in microbial biomass, suggesting the physiological response of the microbial community influenced differences in C and N cycling. At the microbial community scale, we hypothesized that the physiological response of microbial activity to plants could be driven by a shift in community structure, i.e. diversity and composition [5]. Alternatively, if topographic position or soil edaphic characteristics drive microbial community composition and structure, then responses of microbial activity to cropping system are likely due to localized differences in the physiological response of individual species to plant inputs (i.e. physiology or plasticity; [27,28]) rather than community scale changes in composition.

To test the influences of topography, cropping system, and rhizosphere on soil microbial (bacteria, archaea) community structure, we investigated community responses to a fertilized perennial cropping system compared to an annual-based system across a range of edaphic conditions associated with three topographic positions at the Landscape Biomass Project. We used topographic position as a master variable for changes in soil properties because topography affects factors such as soil pH, N availability, moisture regime and plant productivity [29,30]. Given the temporal dynamics of plant effects [16], we sampled whole soil at three times throughout a single growing season and mid-summer in July, around peak aboveground biomass, over two growing seasons. We expected plant effects to be most pronounced mid-summer and in the rhizosphere. Thus, we sampled both whole and rhizosphere soil during mid-summer. Specifically, we hypothesized that 1) α-diversity and community composition (β-diversity, defined here as membership and relative abundance of specific OTUs) of the whole soil would respond more to cropping system than to topographic position, both (a) within a growing season and (b) between years. We predicted greater microbial α-diversity in the perennial cropping system due to increased overall resource supply [31] and greater niche dimensionality as a result of year-round interactions with plants. We also specifically hypothesized that 2) differences in microbial community composition between the annual and perennial cropping systems would be greater in the rhizosphere soil compared to the whole soil. If greater α-diversity (species richness) is due to direct effects of plant inputs, we predicted unique communities would be present in the rhizosphere of specific crop plants (i.e. greater β-diversity or compositional dissimilarity among samples).

## Materials and Methods

### Study site

We conducted this study as part of the Landscape Biomass Project at the Uthe Research & Demonstration Farm in Boone County, Iowa, USA (41°55'N, 93°45'W), where plots of approximately 0.05 ha were established in fall 2008 and first planted in spring 2009. The Uthe Research and Demonstration Farm is owned by the Council for Agricultural Development and managed by Iowa State University, who granted permission for field access and sampling. The field studies did not involve endangered or protected species. Soils at the site are classified as fine-loamy Hapludoll Mollisols and follow a topographic gradient with a slope of approximately 0.5% on the summit and approximately 2.5% on the side slope (back slope and toe slope), as described in detail in Wilson et al. [32]. Soil organic C averaged 17.2 g kg^-1^, total soil N averaged 1.42 g kg^-1^, bulk density averaged 1.57 g cm^-3^, and soil texture averaged 49.4% silt + clay [33]. Notwithstanding similarities in chemistry and root inputs among summit, back slope and toe slope positions, soils from the back slope have significantly lower concentrations of potassium and phosphorus, and soils on the toe slope have significantly greater aggregate geometric mean diameter [33]. Salt-extractable organic C, nitrate, ammonium, pH and water content did not vary with topographic position [26]. Prior to study initiation in 2008, summit and side slopes were managed under a corn-soybean rotation with corn in rotation in 2008. This history of annual-based agriculture may leave a legacy on the microbial community such that changes between microbial communities in the whole soil of annual and perennial cropping systems may be relatively slow (i.e. decades) [34]. Mean monthly air temperatures in 2011 and 2012 did not differ from the long-term mean, but rainfall was extremely variable. Below average rainfall was recorded at the end of summer 2011, followed by a record drought in 2012 [32].

### Experimental design

To assess the impact of cropping system and soil properties on microbial communities, we sampled from a subset of the Landscape Biomass experimental plots, including a no-till annual monoculture (continuous corn, *Zea mays* L.; “annual”) and a perennial monoculture (switchgrass, *Panicum virgatum* L., cv: ‘Cave-In-Rock’; “perennial”), replicated three times at three topographic positions (summit, back slope, and toe slope) following a randomized complete block design (*n* = 3; S1 Fig). Corn was fertilized with N at a rate of 168 kg urea-N ha^-1^ in 2011 and 175 kg urea-N ha^-1^ in 2012, and switchgrass was fertilized at a rate 134 kg urea-N ha^-1^ in 2011 and 135 kg urea-N ha^-1^ in 2012. Both cropping systems received 56 kg P_2_O_5_ ha^-1^ and 112 kg KCl ha^-1^ in 2011. At harvest, maximum aboveground biomass was removed, leaving ~10% of the aboveground biomass from all cropping systems. Refer to Wilson et al. [32] for complete management details.

### Soil sampling

We sampled whole soil, defined as root-free soil collected randomly within plots, and rhizosphere soil, defined as soil adhering to roots from samples collected below a plant. We sampled whole soil by collecting and compositing ten randomly distributed soil cores (2.2 cm in diameter x 15 cm depth) within each plot. We then sieved the soil to 4-mm in the laboratory and subsampled for gravimetric water content and DNA extraction, which we froze at -80°C. To test the effect of soil origin with regard to comparisons between whole and rhizosphere soil, we sampled soil under five randomly chosen plants by taking one core directly under a plant, angled towards the plant stalk. We placed the five cores per plot in sterile bags and transported to the laboratory on ice. Using aseptic technique in the laboratory, we placed the soil on sterilized bench paper and isolated roots from the sample using sterilized tweezers, and removed excess soil from root surfaces with gentle shaking. We sampled the remaining soil that was directly adhered to the root (“rhizosphere soil”) for DNA extraction [9].

We collected whole soil samples in spring after corn emergence and new switchgrass growth (June), mid-summer around peak aboveground biomass (July), and late summer at the beginning of plant senescence (August) in 2011 for a total of 54 composited samples collected in 2011 (3 topographic positions x 2 cropping systems x 3 plots x 3 dates). Based on previous work showing greater microbial activity in the perennial cropping system around peak aboveground biomass in July 2011 [26], we focused sampling efforts to July in 2012 and sampled both whole soil and rhizosphere soil from summit and toe slope positions, for an additional 24 composited samples collected in 2012 (2 topographic positions x 2 cropping systems x 2 soil origins x 3 plots) (S1 Table). To compare extremes of the topographic gradient on rhizosphere communities, we sampled only on the summit and toe slope.

### DNA extraction and 16S rRNA gene sequencing

We extracted DNA from a 0.25 g sub-sample using the PowerSoil-htp 96 Well Soil DNA Isolation Kit (MoBio, Carlsbad, CA, USA), with modifications following the Earth Microbiome Project (EMP; www.earthmicrobiome.org/emp-standard-protocols). We quantified DNA via PicoGreen fluorometry. To assess the α-diversity and β-diversity of the microbial communities, we obtained *16S rRNA* gene sequences following EMP standard protocols [35]. Briefly, we used the 515f/806r primer set to amplify the V4/V5 region of the *16S rRNA* gene and obtained overlapping paired-end 150 base reads using an Illumina MiSeq sequencing system at the Next Generation Sequencing Core (Argonne National Laboratory). We deposited all sequences in the National Center for Biotechnology Information database (BioProject ID: PRJNA248482).

We first merged the raw 150-bp sequence reads using EA-Utils fastq-join [36] and obtained a median merged sequence length of 253-bp. We then processed merged sequences with QIIME v.1.7.0 [37] using default parameters for quality filtering (Phred quality score, Q20) and demultiplexing. We assigned taxonomy by using closed reference UCLUST clustering [38] against the May 2013 release of the Greengenes database filtered at 97% sequence identity [39]. Before downstream analysis, we required that all operational taxonomy units (OTUs) have a count of at least two reads across all samples and rarefied to 6678 sequences per sample to correct for differences in reads across samples. Library size ranged from 6678 to 23,359 sequences per sample with an average of 13,378 sequences per sample.

### Statistical analyses

We quantified microbial communities in the whole soil and rhizosphere in four ways, including three α-diversity measurements (richness, evenness, Shannon’s *H’*) and the Bray-Curtis dissimilarly index to test for differences in community composition, or β-diversity. For each α-diversity measurement (richness, evenness, Shannon’s *H’*), we conducted a full-factorial analysis of variance (ANOVA) on normalized data. We found no difference between results with block as a random effect and with no random effects and present results of the simpler model with no random effects, which allowed us to include Tukey’s HSD to test for multiple comparisons of means. We tested full models with interaction between all main effects and removed non-significant interaction terms from final models. To assess β-diversity, we tested for differences between the Bray-Curtis dissimilarity index using PERMANOVAs with 9999 permutations [40]. Given the large number of OTUs used in multivariate analyses of community composition, we chose PERMANOVAs over parametric tests, such as MANOVA, in order to not violate the assumption of normality. We visualized changes in composition using non-metric multidimensional scaling (NMDS).

For hypothesis 1, we tested for full-factorial differences in α-diversity (ANOVAs on richness, evenness and Shannon’s *H’*) and community composition (PERMANOVAs on Bray-Curtis dissimilarity) in whole soil for samples collected on the three dates in 2011 and between samples collected in July 2011 and 2012. For hypothesis 2, we tested for full-factorial differences in α-diversity and β-diversity between whole and rhizosphere soil (soil origin) collected in July 2012. We also performed ANOVAs and PERMANOVAs with un-rarefied data but found no difference in results compared to rarefied data.

In addition, we summarized our OTUs at the order level and tested for taxa that significantly increased or decreased in rank within communities between whole and rhizosphere soils. We chose to analyze based on rank because this analysis is sensitive to how changes in relative abundance translate to changes in dominance of specific microbial taxa. In this way, changes in rank can catch differences in community structure that are not captured by measurements of community composition. We generated rank-abundance curves for whole and rhizosphere samples within each plot, then calculated differences in rank (Δ_rank_) for microbial orders between whole and rhizosphere soil. We then bootstrapped 95% confidence intervals for Δ_rank_ by subsampling changes in rank position of a specific order within each cropping system and topographic position. When we found significant relationships for a particular microbial order (not containing 0 within the confidence interval), we extended the Δ_rank_ analysis to families and genera within that order.

We performed all analyses in R v.3.0.2 [41] and deposited all R scripts, including libraries used, in an online repository (https://github.com/chnops/Landscape_Biomass_16S; PERMANOVAs, “multivariate_tests”; ANOVAs, “diversity_tests”; delta rank, “delta_rank”).

## Results

### Response in the whole soil

**Fig 1:**
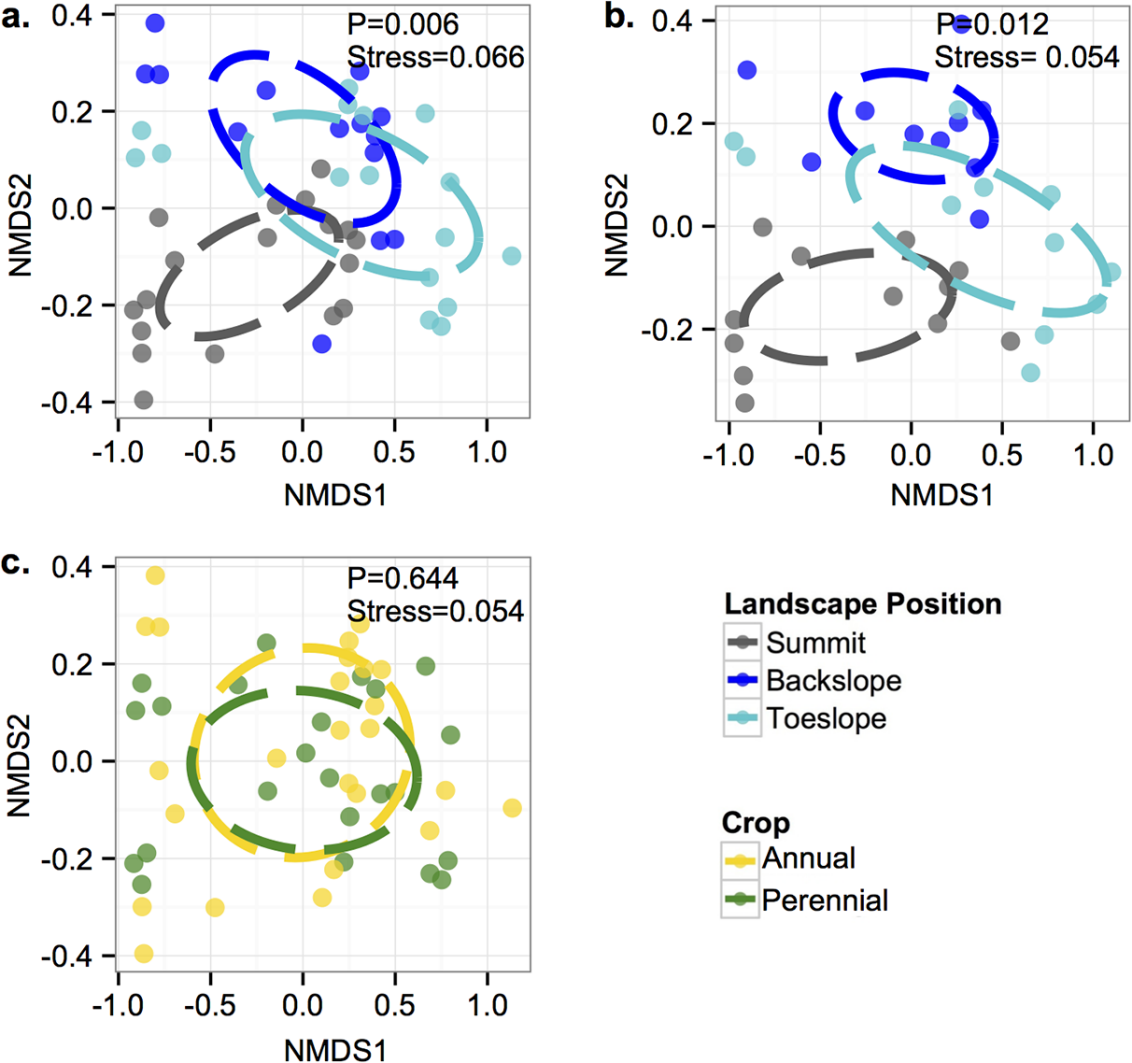
Differences in microbial community composition in the whole soil using the Bray-Curtis dissimilarity index for β-diversity using NMDS. Community composition (a) within the 2011 growing season for each topographic position (3 months x 3 plots x 2 cropping systems), (b) in July 2011 and 2012 for each topographic position (2 years x 3 plots x 2 cropping systems) and (c) between the annual (corn) and perennial (switchgrass) cropping systems (3 months x 3 plots x 3 topographic positions). Using PERMANOVAs, we detected only effects of topographic position for all comparisons. Ellipses represent a centroid of one standard deviation of points.

Within whole soil (hypothesis 1), topography influenced microbial community composition (Bray-Curtis dissimilarity of OTUs) for all analyses. The microbial communities as measured by soil from the summit differed from the back slope and toe slope for all time points in 2011 (Fig 1A; Table 1, hypothesis 1a) and in July 2011 and 2012 (Fig 1B; Table 1, hypothesis 1b). Cropping system did not influence microbial community composition between any of the comparisons tested (Fig 1C; Table 1). There was no detectable effect of time on whole soil microbial community composition, neither within 2011, nor between years in July (Table 1).

**Table 1:** P-values for ANOVA for microbial richness, Shannon’s *H’* and evenness and PERMANOVA for Bray-Curtis dissimilarity

Hypothesis	Source of variation	Richness	Shannon’s *H’*	Evenness	Community Composition*
*1a*: *Whole soil within year*, *June*, *July*, *August 2011*	CS	0.839	0.753	0.800	0.644
	LP	**<0.001**	**<0.001**	**<0.001**	**0.006**
	Month	**0.029**	0.136	0.240	0.836
	LP x CS	NS	NS	NS	NS
	LP x Month	NS	NS	NS	NS
	CS x Month	NS	NS	NS	NS
	LP x CS x Month	NS	NS	NS	NS
*1b*: *Whole soil between years*, *July 2011 & 2012*	CS	**0.014**	0.073	0.177	0.592
	LP	**<0.001**	**<0.001**	**0.001**	**0.012**
	Year	0.206	0.514	0.752	0.663
	LP x CS	NS	NS	NS	NS
	LP x Year	NS	NS	NS	NS
	CS x Year	NS	NS	NS	NS
	LP x CS x Year	NS	NS	NS	NS
*2*: *Whole or rhizosphere soil*, *July 2012*	CS	**0.007**	**<0.001**	**<0.001**	0.268
	LP	**<0.001**	**<0.001**	**<0.001**	**0.011**
	Soil origin	**0.002**	*0*.*070*	0.620	0.184
	LP x CS	NS	NS	NS	NS
	LP x Soil origin	NS	NS	NS	NS
	CS x Soil origin	NS	NS	NS	NS
	LP x CS x Soil origin	NS	NS	NS	NS

LP, topographic position; CS,cropping System; NS, non-signification interaction terms at α > 0.1 and terms were removed from the model

*Community composition evaluated from Bray-Curtis dissimilarity matrices based on OTU abundances.

**Fig 2:**
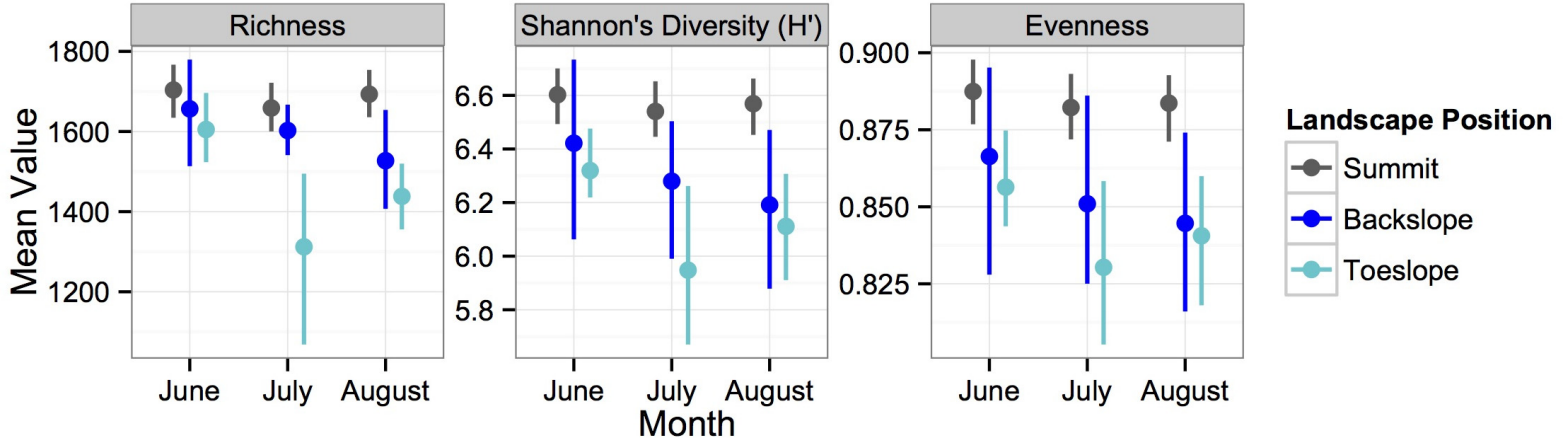
Changes in microbial richness, Shannon’s *H’* and evenness at three topographic positions throughout the 2011 growing season on the summit, back slope and toe slope. These variables were not affected by cropping system or sampling date so values represent means and 95% CI’s of combined annual and perennial treatments at each sampling time.

Microbial α-diversity in the whole soil consistently changed with topographic position. In 2011 and 2012, richness, Shannon’s *H’* and evenness decreased from summit to toe slope (Fig 2; Table 1, hypothesis 1a). We observed greater richness in the whole soil in June compared to July and August in 2011 (Fig 2). There was no difference in microbial richness between years (2011, 2012) for samples taken in July (Table 1, hypothesis 1b).

**Fig 3:**
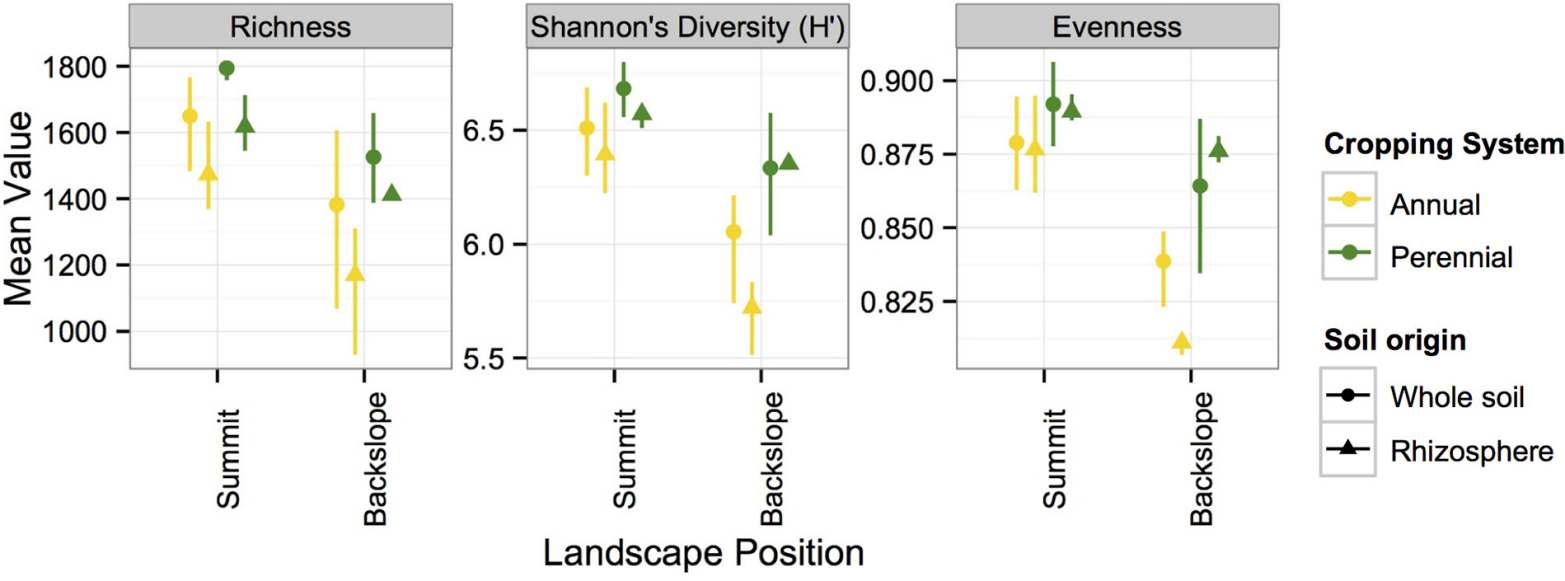
Changes in α-diversity of the microbial communities in July in the whole soil and rhizosphere soil from annual (corn) and perennial (switchgrass) cropping systems at two topographic positions at the Landscape Biomass Project. Means and 95% CI’s are shown.

We did not detect cropping system effects in the whole soil among the three sampling dates in 2011 or within individual sampling dates (Table 1, hypothesis 1a). However, when we compared measurements between years (July 2011, 2012), we detected consistent and significant cropping system effects (Table 1, hypothesis 1b). Our results revealed that cropping system effects on microbial communities were only detectable in July, when plants are fully grown. Specifically, we observed greater Shannon’s *H’* and evenness in the perennial than annual system (Fig 3; Table 1). Similarly, we observed greater microbial richness (P = 0.014) and Shannon’s *H’* (P = 0.073) in the perennial compared to annual cropping system in July 2011 and 2012 (Fig 3).

### Response in the rhizosphere

**Fig 4:**
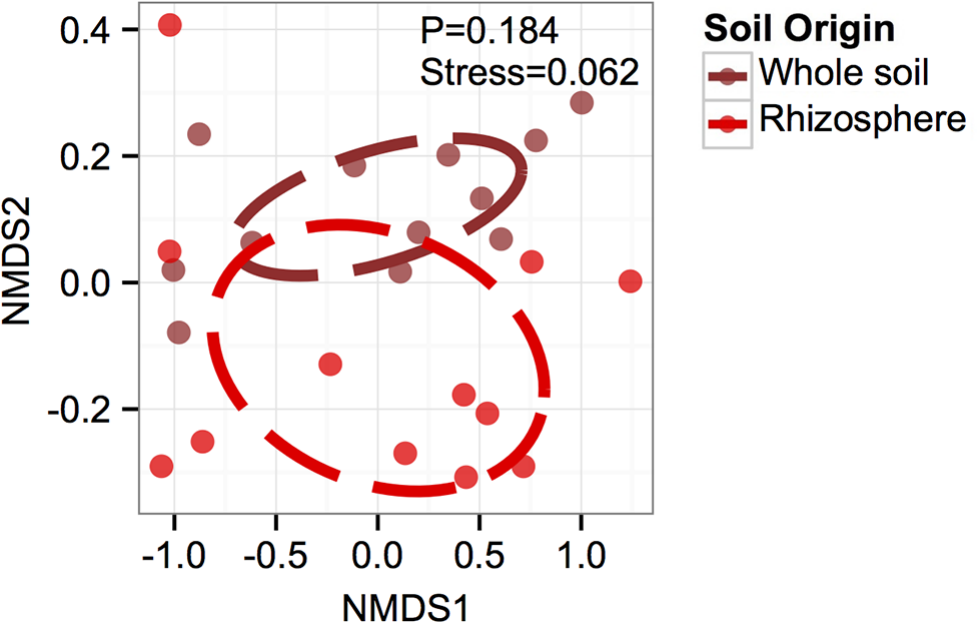
Differences in microbial community composition between whole soil and rhizosphere soil using the Bray-Curtis dissimilarity index for β-diversity (1 date x 3 plots x 2 cropping systems x 2 topographic positions). We detected only effects of topographic position for all comparisons. Ellipses represent a centroid of one standard deviation of points.

With respect to differences between whole and rhizosphere soil (hypothesis 2), topographic position also influenced differences in microbial community composition in 2012 (Table 1). Visualization of microbial community composition via NDMS shows that rhizosphere and whole soil differentiated, though this separation was not statistically significant (P = 0.184; Fig 4). When we compared whole and rhizosphere soils in July 2012, we observed greater microbial richness, Shannon’s *H’* and evenness on the summit than on the toe slope (Fig 3; Table 1). Overall, the rhizosphere was less rich (P = 0.002) and less diverse (Shannon’s *H’*; P = 0.07) than the whole soil with no detectable changes in evenness (P = 0.620).

**Fig 5:**
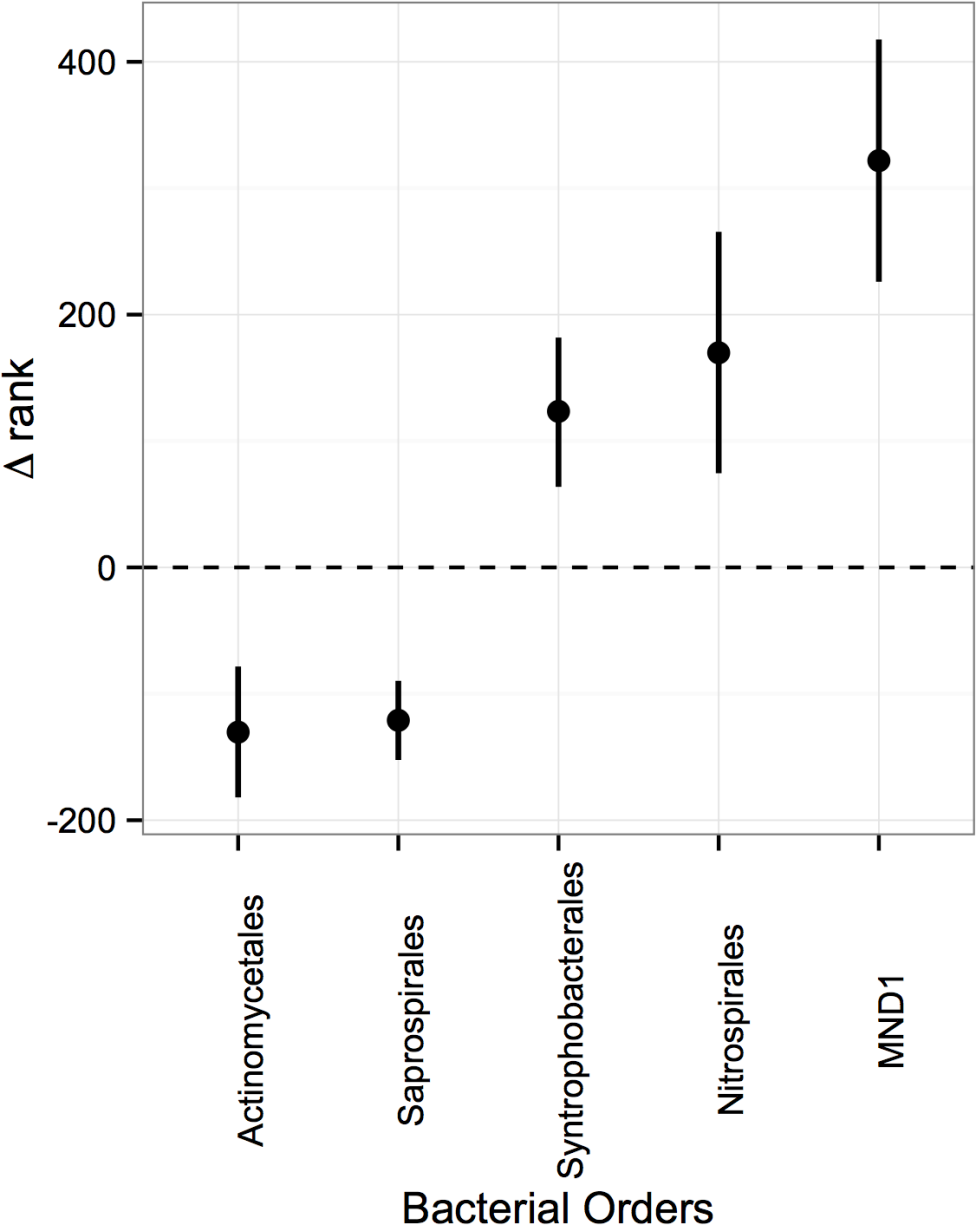
Change in rank abundance from whole to rhizosphere soil for Actinomycetales, Saprospirales, Syntrophobacterales, Nitrospirales, and MND1 bacterial orders. Positive values indicate the order has a higher rank in the whole soil and negative values indicate the order has a higher rank in the rhizosphere soil. Error bars represent 95% CI’s.

Of the 243 orders captured in this study, we used differences in rank (Δ_rank)_ to detect shifts in the relative abundance of 16 bacterial orders between whole and rhizosphere soil (S2 Table). Of these, we observed consistent enrichment of Actinomycetales and Saprospirales in the rhizosphere soil on both summit and toe slope and of Nitrospirales, Syntrophobacterales and MND1 in the whole soil on both summit and toe slope (Fig 5). For two orders enriched in the rhizosphere, Actinomycetales and Saprospirales, we also observed greater abundance of specific families and genera in the rhizosphere. Within the Actinomycetales, we observed a greater abundance of Actinospicaceae, Frankiaceae, and Cellulomonadaceae, especially genera *Cellulomonas*, in the rhizosphere. Within the Saprospirales, we observed a greater abundance in the rhizosphere of bacteria within the family Chitinophagaceae. In contrast, we observed equal enrichment of families and genera within Nitrospirales, Syntrophobacterales and MND1 in the rhizosphere and whole soil.

## Discussion

We sought to investigate the role of cropping system, rhizosphere effects and topographic position in shaping microbial communities under field conditions. Based on previous results demonstrating differences in microbial activity between cropping systems but not topographic positions [26], we hypothesized that microbial α-diversity and β-diversity in the whole soil would be primarily shaped by cropping system (hypothesis 1). Consistent with previously published work on microbial activity at this site [26], we only detected cropping system effects on the microbial α-diversity mid summer in July, coincident with peak aboveground biomass. Our finding of greater α-diversity without a change in composition indicates that the dominant taxa are similar between cropping systems, but microbial communities under perennials contain more less-abundant taxa relative to the annual cropping system. Further, because we only detected cropping system effects around peak aboveground biomass, our data suggest that plant phenological traits, such as timing of rhizodeposition and fine root inputs, stimulated these shifts in less abundant taxa [16,42–44]. Considering the remarkable stability of microbial composition across time, even with a drought in 2012, these shifts in α-diversity (richness, Shannon’s *H’*, evenness) may account for previously observed temporal changes in microbial enzyme activity, respiration and mineralization [26].

In contrast to hypothesis 1, microbial community composition was most strongly and consistently affected by topographic position, despite no differences in pH. Generally, we observed distinct microbial communities on the summit with greater α-diversity than those on the back slope and top slope positions. While most soil physiochemical characteristics do not differ among the three topographic positions, previous research at this site has shown that the summit has higher concentrations of potassium and phosphorus than the back slope and toe slope [33]. Although not often considered in studies of microbial biogeography, phosphorus and potassium concentrations correlate with the abundance of microbial phyla in agricultural fields [45] and, thus, may be important drivers of microbial communities when N-limitation is relieved through the addition of fertilizer. We recognize that edaphic factors often co-vary such that topographic position results in uncharacterized effects; additional work is needed to test the effects of individual or suites of edaphic factors on microbial community structure.

Contrary to hypothesis 2, the rhizosphere of the perennial and annual plant did not uniquely shape microbial communities from the whole soil (i.e. no soil origin x cropping system interaction). Differences between rhizosphere and whole soil were not cropping system-specific. Rhizosphere communities were less rich than whole soil communities, indicating a general enrichment of root-associated taxa in rhizosphere soil. Together with less rich microbial communities, we observed consistent changes in relative abundance of a small number of the most dominant microbial orders, irrespective of cropping system and topographic gradient. In rhizosphere soils, we observed a greater abundance of the ubiquitous Actinomycetales, a filamentous group of bacteria that can promote plant growth [46,47], and Saprospirales, previously shown in high alpine systems to be predictive of plant cover, β-Glucosidase activity, soil water, dissolved organic C, and pH [48]. Enrichment of specific families and genera within these orders suggests an importance of plant cell wall material (i.e. cellulose degraders Cellulomonadaceae) and fungal cell wall material,(i.e. chitin degraders Chitinophagaceae) for the selection of organisms to the rhizosphere. The fact that shifts in these taxa were not cropping system-specific suggests that they may be more general rhizosphere colonizers.

Unlike previous findings suggesting Nitrospira are also rhizosphere generalists in unmanaged systems [49,50], we observed Nitrospirales in less abundance in the rhizosphere compared to whole agricultural soil. Given findings by Turner et al. [27], who found depletion of Nitrospira in the rhizosphere of a legume (pea) relative to the whole soil, the depletion of Nitrospirales in the rhizosphere that we observed might be attributable to N fertilization. Under N limiting conditions, N-rich microsites can select for both roots and nitrifiers [51]. When N is not limiting for plants and/or nitrifiers, however, other selective forces may take precedent over their distribution and co-occurrence, implying that resource needs by plants can lead to the enrichment or depletion of some taxa in the rhizosphere [52]. Selective conditions in the whole soil, such as compaction from lack of root penetration, may also lead to taxon enrichment or depletion, as demonstrated by the greater abundance of strict anaerobes like Syntrophobacterales in whole soil compared to the rhizosphere soil.

Our results support environmental filtering for the microbial community by the soil conditions, including general plant effects related to time of sampling but not plant-specific effects within the rhizosphere. The lack of observed effect of cropping system or soil origin (rhizosphere vs. whole soil) on overall microbial community composition stands in contrast to the current paradigm that plant-specific traits influence environmental filtering of rhizosphere communities [27,52,53]. Specific to our system, previous field studies that have found differences in microbial community composition in the whole soil of perennial bioenergy cropping systems [54,55]. Although the legacy of conventional, annual-based agriculture may have limited cropping system effects to overall community composition [56–58], it has not prevented changes in microbial communities in response to perennial bioenergy cropping systems in similar soils in the Corn Belt of the United States [54,55,59]. The most unique attribute of the perennial cropping system in this study is N fertilization, which is not part of perennial cropping systems in other studies. Indeed, recent work suggests plant N status drives microbial community structure in the rhizosphere [52]. Therefore, while other factors can not be ruled out, N fertilization at this site may have masked plant-specific effects on microbial community composition [20,21,60], suggesting soil nutrient amendments hinder crop plants’ ability to directly influence rhizosphere communities.

## Conclusions

Topographic position was the strongest and most consistent driver of microbial community composition (β-diversity) in our system, both within whole and rhizosphere soils. Soil properties associated with topography, therefore, had precedent over plant effects in shaping microbial community composition in these soils. Given that both cropping systems were fertilized, N fertilization may also preclude cropping system-specific microbial response as demonstrated by similar microbial communities in annual and perennial systems.

Results presented here also provide evidence that cropping system shape a small subset of organisms in the whole soil at specific times in the growing season. Given that previously documented changes in microbial activity [26] coincided with our observed shifts in diversity, we hypothesize that the activity of less abundance organisms may provide novel insights into composition-function relationships of soil microbial communities. Further, factors shaping rhizosphere communities in this system are related to general plant effects, but not plant-specific effects. We identified lifestyles (i.e. filamentous) and metabolic capabilities (i.e. cellulose and chitin degradation, but not nitrate-oxidation) associated with the rhizosphere of these fertilized crops. Overall, this work demonstrates the overarching importance of edaphic factors for shaping microbial community composition and the importance of plant effects and phenology for determining changes in relative abundance of specific microbial taxa.

## Supporting Information

S1 Fig Experimental design of five cropping systems and five topographic positions at the Landscape Biomass Project, USA. Whole soil samples were taken only from the perennial switchgrass and annual corn cropping systems on the summit, back slope and toe slope, denoted in bold and by asterisks. Rhizosphere soil was sampled only from summit and toe slope positions. Modified from Wilson et al. 2014.
(DOCX)

S1 Table Soil sampling details for the experiment conducted at the Landscape Biomass Project, USA. (DOCX)

S2 Table Changes in the relative abundance of microbial orders between whole and rhizosphere soil on the summit and toe slope positions at the Landscape Biomass Project, USA. Soil samples were taken mid-summer, at peak biomass in 2012.
(DOCX)
