# Supplementary material for: Environmental Filtering of Microbial Communities in Agricultural Soil Shifts with Crop Growth
Source: PLoS One. 2015 Jul 30;10(7):e0134345. doi: 10.1371/journal.pone.0134345 (PMC4520589; doi:10.1371/journal.pone.0134345)

**Figure S1.** Experimental design of five cropping systems and five topographic positions at the Landscape Biomass Project, USA. Whole soil samples were taken only from the perennial switchgrass and annual corn cropping systems on the summit, back slope and toe slope, denoted in bold and by asterisks. Rhizosphere soil was sampled only from summit and toe slope positions. Modified from Wilson et al. 2014.


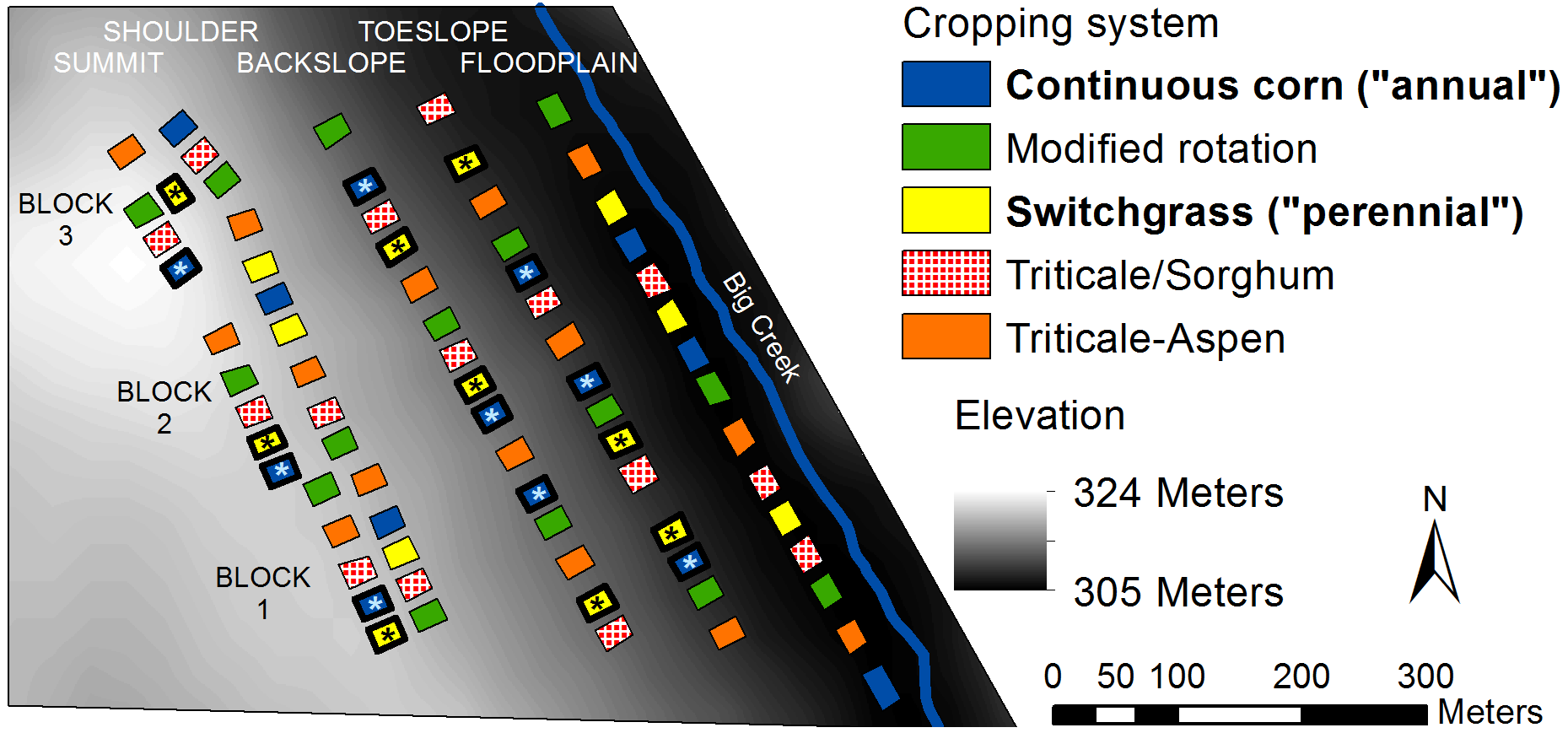

Supplement: S1 Fig — Whole soil samples were taken only from the perennial switchgrass and annual corn cropping systems on the summit, back slope and toe slope, denoted in bold and by asterisks. Rhizosphere soil was sampled only from summit and toe slope positions. Modified from Wilson et al. 2014. (DOCX) [file pone.0134345.s001.docx]
