# Supplementary material for: Environmental Filtering of Microbial Communities in Agricultural Soil Shifts with Crop Growth
Source: PLoS One. 2015 Jul 30;10(7):e0134345. doi: 10.1371/journal.pone.0134345 (PMC4520589; doi:10.1371/journal.pone.0134345)
Supplement: S1 Table — (DOCX) [file pone.0134345.s002.docx]

**Table S1**. Soil sampling details for the experiment conducted at the Landscape Biomass Project, USA.

| **Sampling** | **Year** | **Date** | **Soil sample*** | **Location** |
| --- | --- | --- | --- | --- |
| Spring | 2011 | June 6 | Whole | Summit, back slope, toe slope |
| Peak aboveground biomass | 2011 | July 13 | Whole | Summit, back slope, toe slope |
| Late summer | 2011 | August 23 | Whole | Summit, back slope, toe slope |
| Peak aboveground biomass | 2012 | July 11 | Whole | Summit, back slope, toe slope |
| Peak aboveground biomass | 2012 |  | Rhizosphere | Summit, toe slope |

*Whole soil refers to soil not adhered to a root surface while rhizosphere soil refers to soil adhered to a root surface.
