# Supplementary material for: Environmental Filtering of Microbial Communities in Agricultural Soil Shifts with Crop Growth
Source: PLoS One. 2015 Jul 30;10(7):e0134345. doi: 10.1371/journal.pone.0134345 (PMC4520589; doi:10.1371/journal.pone.0134345)
Supplement: S2 Table — Soil samples were taken mid-summer, at peak biomass in 2012. (DOCX) [file pone.0134345.s003.docx]

**Table S2.** Changes in the relative abundance of microbial orders between whole and rhizosphere soil on the summit and toe slope positions at the Landscape Biomass Project, USA. Soil samples were taken mid-summer, at peak biomass in 2012.

| **Summit** | | **Toe slope** | |
| --- | --- | --- | --- |
| **Rhizosphere>Whole** | **Rhizosphere<Whole** | **Rhizosphere>Whole** | **Rhizosphere<Whole** |
| Saprospirales* | 32-20 | Saprospirales* | BPC015 |
| Actinomycetales* | A21b | Actinomycetales* | CCM11a |
| Cytophagales | Defulfuromonadales | Bdellovibrionales | Cenarchaeales |
| JG30-KF-CM45 | FW68 | Burkholderiales | CFB-26 |
| Methylphilales | Gaiellales | Ellin7246 | Chtoniobacterales |
| RB41 | MKC10 | Opitutales | Ellin6513 |
|  | MND1* | Rhizobiales | Enteromicrobiales |
|  | Nitrospirales* | Sphingomicrobiales | FAC87 |
|  | Oscillatoriales | Streptophyta | LLb |
|  | Rhodospirillales |  | MND1* |
|  | Rubrobacterales |  | MVS-40 |
|  | Solirubrobacterales |  | Nitrospirales* |
|  | Syntrophobacterales* |  | NRP-J |
|  |  |  | S0208 |
|  |  |  | Spirobacillales |
|  |  |  | Syntrophobacterales* |

*Microbial orders with changes on both summit and toe slope
